# Prognostic Value of Cardiovascular Testing in Asymptomatic Patients With a History of Cardiovascular Disease: A Review of Contemporary Medical Literature

**DOI:** 10.7759/cureus.16892

**Published:** 2021-08-04

**Authors:** Kelechi E Emmanuel, Mahmoud Nassar, Nso Nso

**Affiliations:** 1 Internal Medicine, University of Pittsburgh Medical Center Pinnacle, Harrisburg, USA; 2 Internal Medicine, Icahn School of Medicine at Mount Sinai/New York City (NYC) Health+Hospitals Queens, New York, USA; 3 Internal Medicine, Icahn School of Medicine at Mount Sinai/New York City (NYC) Health+Hospitals, New York, USA

**Keywords:** cardiology, cardiovascular screening, peripheral artery disease, cardiac catheterization, coronary artery calcium scoring, glycohemoglobin, ankle-brachial index, coronary artery angiography, magnetic resonance angiography, quantitative magnetic resonance angiography

## Abstract

The cardiac stress testing, carotid duplex, coronary artery calcium (CAC) scoring, myocardial perfusion imaging, coronary angiography, C-reactive protein (CRP), glycated hemoglobin (HbA1C), total serum cholesterol, duplex ultrasonography, digital subtraction angiography, magnetic resonance angiography (MRA), computed tomography angiography (CTA), digital subtraction angiography, and ankle-brachial index (ABI) independently predict the risks and prognostic outcomes in asymptomatic cardiovascular disease (CVD) patients. The peripheral artery disease (PAD) screening guides the diagnosis, management, and prognosis of hemodynamically significant arterial stenosis, calcification, and malignant hypertension in patients with CVD without symptoms. The 79% sensitivity and 96% specificity of ABI screening, 90% sensitivity and 97% specificity of MRA, and 95% sensitivity and 50% specificity of CTA for tracking arterial occlusion indicate the high prognostic value of these tests in the setting of CVD. The 85% specificity and 60-70% sensitivity of cardiac stress testing substantiate its suitability to determine asymptomatic CVD prognosis related to myocardial ischemia, heart failure, multivessel disease, and unstable angina. The carotid duplex ultrasound potentially identifies long-term mortality, stroke, atherosclerosis, plaque instability, and angiographic stenosis among asymptomatic CVD patients with 94% specificity and 90% sensitivity. The CAC scoring has a positive predictive value (PPV) of 45.7% for identifying aortic valve calcium and PPV of 79.3% for tracking thoracic artery calcium. The medical literature provides substantial evidence concerning the validity, reliability, and prognostic value of cardiovascular testing for asymptomatic patients. Future studies are needed to undertake detailed assessments of benefits versus adverse outcomes associated with the prospective scaling (of cardiovascular testing) across asymptomatic CVD patients.

## Introduction and background

More than one-third of mortality in the United States reportedly occurs under the impact of cardiovascular disease (CVD) that majorly includes aortic atherosclerosis, peripheral artery disease (PAD), cerebrovascular disease, and coronary heart disease/coronary artery disease (CAD) [1]. The sustained reduction in myocardial perfusion often results in CAD that triggers ischemia-induced angina and myocardial infarction (MI). Approximately one-third to half of CVD patients experience MI, heart failure, or angina [2]. Ischemic heart disease potentially elevates the disability-adjusted life years of CVD patients, while its potential risk factors include high body mass index, hypercholesterolemia, and high blood pressure [3]. Approximately 422.7 million patients across the globe develop CVD every year, but medical literature records marked variations in its age-adjusted incidence and prevalence [4]. The global estimates affirm the ever-increasing burden of CVD that may acquire a prevalence of 1,917 cases per hundred thousand individuals until 2030 [5].

Cardiovascular testing is the key to improving and optimizing prophylaxis and treatment in the setting of CVD. The assessment of total serum cholesterol and glycated hemoglobin (HbA1c) levels is paramount in investigating the CVD risk levels of asymptomatic patients [6]. Coronary artery calcium (CAC) scoring also helps stratify the CVD risk in 10-20% of individuals without symptoms and cardiovascular assessments including, ankle-brachial index (ABI) and quantification of carotid plaque/carotid-intima media thickness identify the predisposition of asymptomatic patients to cardiovascular events. The high sensitivity C-reactive protein (CRP) test assists in tracking the cardiovascular health of asymptomatic (intermediate-risk) patients with/without CVD [7]. Fifteen percent of CVD patients (with a reported five-year risk) benefit from a stress myocardial perfusion scan (MPS) that helps determine their cardiac function and prognostic outcomes. Other specific cardiovascular tests include screening for PAD, computerized tomography (CT), coronary angiography, carotid duplex, MPS, stress echocardiography/cardiac stress testing, and coronary angiography [8]. Duke Treadmill Score obtained through the electrocardiogram (ECG/EKG) exercise testing also guides the risk stratification of patients suspected to have CAD [9].

The conflicting opinions regarding the diagnostic efficacy of cardiovascular testing (in the setting of asymptomatic CVD) barricade the optimization of medical therapies [10]. The clinical studies continue to explore the potential of cardiovascular testing in predicting the risk of ischemia or the scope of recanalization. The current perspectives regarding cardiovascular testing also do not substantiate its precision in determining the cardiovascular risk factors among asymptomatic patients with comorbid conditions [11]. Furthermore, the potential of cardiovascular testing (to identify the CVD risk factors among asymptomatic patients with a clinical history of hypertension, smoking, and diabetes mellitus) also requires further research and investigation [12]. This review paper accordingly evaluates the current body of evidence concerning the diagnostic efficacy of cardiovascular testing in prognosticating and stratifying the CVD risk factors among asymptomatic patients. 

## Review

PAD screening

The cardiovascular testing for PAD relies on several diagnostic interventions, including ABI, computed tomography angiography (CTA), magnetic resonance angiography (MRA), digital subtraction angiography, and duplex ultrasonography [13]. ABI measures the ratio of systolic/diastolic blood pressures across the ankle/brachial artery. The ABI cut-off value of 0.9 or less helps determine age-appropriate CVD risk factors across asymptomatic adult populations. ABI can be used as a risk assessment tool in asymptomatic patients with elevated cardiovascular risk factors such as smokers or patients with a previous history of CAD. The elevated ABI values in these asymptomatic patients reveal increased predisposition to major adverse cardiovascular events and to malignant hypertension and diabetes while the ABI range of 1.30-1.4 correlates with the reduced possibility of incompressible vasculature [14]. The ABI screening intervention exhibits 96% specificity and 79% sensitivity in diagnosing lower extremity peripheral arterial disease. Higher ABI scores clinically correlate with critical limb ischemia in asymptomatic diabetic patients with risks up to 2.4 times more. PAD-positive asymptomatic CVD patients experience a 55% prevalence of hypertriglyceridemia, hyperlipidemia, and high LDL/low HDL levels [15]. ABI values (when correlated with past medical history) help predict the risk of stroke in asymptomatic CVD patients. MRA effectively tracks hemodynamically significant stenosis in asymptomatic CVD patients with 97% specificity and 90% sensitivity [16]. High-quality images from gadolinium-enhanced three-dimensional MRA help identify strictures, narrowing, and blockage in lower extremity arteries of asymptomatic patients with CVD while high-resolution/multi-detector CTA tracks vascular occlusions/arterial stenosis in asymptomatic CVD patients with 50% specificity and 95% sensitivity [16]. The high spatial resolution of CTA images helps visualize stent grafts/cardiovascular stents, metallic implants, and calcification. CTA also visualizes anatomic structures and soft tissues through volumetric acquisition from multi-detector row scanners. Duplex ultrasonography effectively identifies lower extremity arterial stenosis/occlusion in asymptomatic CVD patients. The velocity parameter derived from pulsed-wave doppler/color flow doppler helps evaluate the location and severity of arterial occlusion. Duplex ultrasonography also delineates arterial occlusion and stenosis separately in the setting of CVD [16]. It further produces two-dimensional figures of the lumen and wall of the arteries and displays the structure and characteristics of atheromatous plaques in asymptomatic CVD patients. Digital subtraction angiography utilizes intravascular ultrasonography to visualize blood vessel wall layers and pressure gradients [16]. However, its utilization in asymptomatic patients remains questionable due to the potential risks concerning catheterization/vascular access, iodinated contrast agents, and ionizing radiation.

Cardiac stress testing 

Cardiac stress testing tracks and identifies CVD-related risks in asymptomatic patients with 85% specificity and 60-70% sensitivity [17]. The electrocardiogram stress test effectively determines ischemic changes in asymptomatic CVD patients. It also helps evaluate the severity and prognostic outcomes in asymptomatic patients with a known history of CAD. Medical literature reveals 77% pooled specificity and 68% sensitivity of exercise stress electrocardiography in evaluating CAD in asymptomatic patients. In this patient population, outcomes of cardiac stress testing guide the assessment of latent CAD, congestive heart failure, arrhythmias, and congenital heart disease. It also predicts the risk of multi-vessel disease and unstable angina. Furthermore, the results of cardiac stress testing determine the need for exercise echocardiography/myocardial perfusion imaging and other follow-up assessments for high/intermediate-risk CVD patients. Clinical correlation between baseline electrocardiogram findings (indicating preserved resting left ventricular function) and outcomes from cardiac stress testing informs the prognostication and cardiac tissue re-vascularization requirements in asymptomatic CVD cases [18,19]. Cardiac stress testing also predicts mortality rates in asymptomatic CVD patients. Exercise stress test results showing less than 1 mm ST depression in accordance with the three-minutes Bruce protocol correlates with a CVD mortality rate of less than 1%; while a 5% annual CVD mortality rate correlates with ST depression (≥1 mm) in scenarios where the asymptomatic patients fail to accomplish the first phase of the Bruce protocol [19]. It is important to mention that chronotropic incompetence of CVD patients in terms of achieving 85% age-adjusted heart rate via cardiac stress testing determines their risk of all-cause mortality and progressive heart failure [20]. In conclusion, results concerning cardiac stress testing in asymptomatic patients as seen in ST-segment depression levels and reduction in chronotropic indices (recorded through EKG stress test) provide an insight into the risk of cardiovascular mortality among asymptomatic CVD patients.

Carotid duplex

The carotid duplex ultrasound helps evaluate the risk of atherosclerotic disease and transient ischemic attack in asymptomatic patients by assessing their carotid artery stenosis [21]. This test tracks carotid stenosis among 70-90% of asymptomatic patients with a clinical history of CVD. The criteria for evaluating carotid stenosis in asymptomatic patients are the occurrence of arterial stricture without a known focal neurological deficit or past medical history of cerebrovascular accident/stroke. More than 50% narrowing (of the arterial lumen) accounts for less than 1% annual stroke prevalence in asymptomatic CVD patients [22]. Luminal narrowing above 80% in asymptomatic patients alternatively predicts their 5% yearly predisposition to stroke and other cardiovascular events. The thickening of intima-media determined via carotid duplex also facilitates CVD prognostication in asymptomatic scenarios. There is now an advocation to use the results of a carotid duplex in anticipating long-term mortality in neurologically asymptomatic diabetic patients with CVD/carotid arteriosclerosis [23]. This is because the results obtained from carotid duplex screening have a high validity and reliability making it a preferred diagnostic modality for evaluating the risk and prognosis of atherosclerosis in the setting of CVD [24]. According to Mughal et al., carotid duplex predicts the risk of ischemic stroke among 15-20% of asymptomatic CVD patients [25]. CVD prognosis assessment potential of carotid duplex reciprocates with the gender and smoking history of these asymptomatic patients. Carotid duplex findings also indicate the plaque instability level that helps determine the risk of ischemic complications and the possible need for invasive therapies in the setting of CVD. The carotid duplex modality helps evaluate the characteristics of atheromatous lesions that predict the risk of 70% internal carotid artery occlusion in asymptomatic patients with CVD. It also predicts the risk of non-fatal stroke, stroke rate, MI, and cardiovascular death at one year in the context of CVD. Results of carotid duplex also indicate the predisposition of asymptomatic PAD patients for cerebrovascular complications before and after receiving invasive therapies [26]. According to published medical literature, there is a 94% specificity and 90% sensitivity of carotid duplex ultrasound in evaluating the risk of angiographic stenosis in more than 70% of CVD patients, and localization of carotid bruit by carotid duplex measure in asymptomatic CVD patients helps determine their revascularization interventions and other therapies for carotid artery disease management [27].

CAC scoring

The diagnostic assessment of coronary atherosclerosis relies on the systematic use of CAC scoring. The risk stratification capacity (of the CAC scoring method) helps predict the prognostic outcomes in asymptomatic CVD patients. A CAC score of 1000 or above informs the treatment modalities for patients with CVD. CAC testing has a positive predictive value (PPV) of 45.7% for identifying aortic valve calcium and PPV of 79.3% for tracking thoracic artery calcium. A high CAC score within asymptomatic CVD patients of age group 30-49 years indicates their mortality risk and requirement for aggressive treatments and also determines the prognostic outcomes in asymptomatic patients with a family history of CVD [28]. The CAC score of zero in asymptomatic patients minimizes their over-prescription while de-risking them and predicting their recovery rate. The CAC score of double-zero (at five years and baseline) correlates with a 1.4-1.8% (10-5) year risk of poor prognosis in the setting of CVD. The CAC scoring also predicts coronary heart disease outcomes and CVD-related deaths among asymptomatic patients. It also predicts age-adjusted all-cause mortality and major adverse cardiovascular events (including stress-induced myocardial ischemia) among asymptomatic diabetic CVD patients [29]. The statistical risk reclassification guided by CAC assists in segregating high-risk and low-risk (asymptomatic) CVD patients. The net reclassification index also informs preventive therapies warranted to improve the prognostic outcomes of CVD patients. The attenuation-oriented hybrid scanning guided by positron emission tomography (PET) myocardial perfusion imaging, single-photon emission computed tomography (SPECT), and CAC scoring effectively predicts risk factors/prognostic outcomes and strengthens the preventive decision-making for asymptomatic CVD patients [30]. Clinical studies advocate for the potential of CAC scoring in evaluating the severity of CAD among asymptomatic elderly male patients with internal carotid artery stenosis, PAD, and dyslipidemia [31]. Diagnostic assessment of subclinical atherosclerosis (in asymptomatic patients with first-degree relatives with CAD) guided by CAC scoring informs their treatment modalities. CAC scores also guide the prognostication of asymptomatic patients with a Framingham Risk Score of less than 10% and further guides the 10-year atherosclerotic CVD risk assessment in asymptomatic patients of the age range 40-75 years [32,33]. This scan also informs the lifestyle management approaches (in the setting of CVD) and CAC scores between 0 and 300 guide the antiplatelet therapies for the asymptomatic atherosclerotic CVD patients [34].

MPS/imaging

MPS deploys radiotracers directed by SPECT/PET to produce high-quality images (of the myocardial cells) constructed by the interactions between iodide crystals and photons [35]. These images reflect the vascular supply of the heart muscle that helps analyze coronary health and risk factors of CVD patients. The concomitant use of EKG stress test with myocardial perfusion imaging helps evaluate the risk factors and prognosis (of asymptomatic CVD patients). The calculation of coronary flow reserve via MPS outcomes helps predict the risk of cardiac death and progression of coronary microvascular/diffuse disease and focal stenosis in the setting of CVD. In this imaging study, the impairment in coronary flow reserve indicates a 4.9-fold risk of cardiovascular mortality in asymptomatic CVD patients [10]. The MPS is the first-line diagnostic modality that measures the aggregate impact of endothelium/small vessel/coronary artery pathology. It also assists in predicting the yearly risk of MI and all-cause mortality prevalence among asymptomatic patients [36]. MPS results also inform the risk stratification and long-term management of acute coronary syndromes and acute MI. Normal MPS findings concerning asymptomatic CVD patients indicate their reduced (less than 1%) predisposition for non-fatal MI and cardiovascular death, while abnormal MPS scores alternatively reveal a high risk for ischemic perfusion defects and multi-vessel disease incidence. The MPS scores concerning asymptomatic patients with a past medical history of MI indicate their risk of left ventricular ejection fraction reduction and myocardial ischemia. They also help categorize asymptomatic CVD patients based on their high, intermediate, or low risk for cardiovascular complications. They guide the assessment of dysfunctional (viable) hibernating myocardium and myocardial scarring, indicating a fixed defect [37]. The myocardial perfusion imaging facilitates the prognostication/prediction of future coronary events among asymptomatic CVD patients with 85-90% sensitivity. This elevated sensitivity/specificity of the MPS scan helps minimize false negative/false positive results concerning asymptomatic CVD patients. MPS-guided clinical parameters that determine the risk (of future coronary episodes) among asymptomatic CAD patients include, left ventricular ejection fraction, stress-induced ventricular dilation, thallium uptake level across lungs, and severity/extent (of exercise-induced ischemia). The abnormal MPS findings attribute to 6.7% of yearly MI/cardiac death episodes among CVD patients [38]. Furthermore, the atherosclerosis assessment capacity of myocardial perfusion imaging makes this test a robust prognostication tool in CAD scenarios [39].

Coronary angiography

CT-guided coronary angiography effectively detects more than 50% coronary artery stenosis cases with 83% specificity and 95% sensitivity [40]. It also diagnoses asymptomatic coronary heart disease among individuals below 40 years of age. Coronary angiography findings prognosticate mortality and progressive development of non-obstructive plaques in 50% of the patients with coronary artery stenosis. It also tracks obstructive plaques in the left circumflex, right coronary, and left anterior descending arteries. The potential of coronary angiography to elaborate on fractional flow reserve and functional significance of coronary stenosis makes it a preferable prognostication tool for asymptomatic patients [41]. Findings from elective angiography for asymptomatic CVD patients conclusively predict their stenosis distribution patterns and occlusions that obstruct more than 70% of their left main coronary artery [42]. Coronary angiography also detects major adverse cardiac events (MACE) that help determine the long/mid-term prognosis of asymptomatic patients. This diagnostic modality predicts the risk of MI/non-ST segment elevation MI (NSTEMI), cardiovascular mortality, non-obstructive CAD, and unstable angina/acute coronary syndrome in the setting of CVD [43]. Findings from coronary angiography also inform the need for revascularization and hospitalization among asymptomatic CVD patients. CTA findings guide the prognostication of aortic disease, stroke, and heart failure in the setting of asymptomatic CVD. They help determine the risk of all-cause mortality, severe CAD, arrhythmic/valvular heart disease, congestive heart failure, and atherosclerotic coronary vascular disease in asymptomatic patients [44]. Medical literature confirms the potential of coronary angiography in tracking 53-74% prevalence of 50-100% coronary artery stenosis among asymptomatic patients [45]. It has a 0.74 PPV for coronary stenoses manifested with ≥30% luminal narrowing. Findings from coronary angiography inform the need for statin therapies for these patients to minimize their predisposition for cardio-metabolic adversities and also reveal the variability in cardiovascular risk/prognosis in asymptomatic patients with a medical history of diabetes [46]. Coronary angiography also predicts the prospective requirement of percutaneous coronary intervention (PCI) for asymptomatic CVD patients with 50-80% arterial stenosis [47]. The clinical correlation of coronary angiography findings with smoking history, systolic blood pressure, cholesterol levels, ethnicity, and age of the asymptomatic CVD patients further guides their prognosis and risk stratification [48].

C-reactive protein

CRP is an evidence-based prognostic marker of CVD complications. CRP levels of 10 mg/L or above indicate the long-term risk of cardiovascular adversities requiring preventive and prophylactic management [49]. These findings also assist in predicting all-cause mortality among 60% of CVD patients. They further guide the prognostication of asymptomatic patients based on their risk of stroke and coronary episodes. CRP levels have also been shown to predict the CVD outcomes/prognosis/risk factors in asymptomatic incident diabetes patients. Clinical correlation of elevated high sensitivity CRP levels with the medical history of obesity, dyslipidemia, diabetes/hyperglycemia, hypertension, and hypercholesterolemia guides the prognostication of MACE and cardiovascular mortality across asymptomatic CVD patients. Concomitant assessment of interleukin 1-beta, tumor necrosis factor-alpha, interleukin-6, and intermediate/very low/low/oxidized low-density lipoproteins further predicts long-term (vulnerable plaque-related) complications concerning stroke/heart attack in asymptomatic patients [50]. The CRP value of greater than 3 mg/L independently predicts intermediate/high risk of cardiovascular episodes in asymptomatic patients after adjusting their CAC and Framingham Risk Scores [51,52].

Total serum cholesterol and HbA1c

Total serum cholesterol level clinically correlates with the high predisposition of asymptomatic CVD patients for cerebrovascular disease and ischemic heart disease complications [53]. It independently predicts all-cause mortality, vascular death, and serious adverse events among asymptomatic patients with CVD [54]. The reductions in total serum cholesterol below 200 mg/dL correlate with higher survival rates and quality-adjusted life years. Mortality risk assessment also reciprocates with the baseline cholesterol levels of the CVD patients. Correlation of comorbidity index, income status, smoking status, alcohol dependence, physical activity, body mass index, gender, and age with cholesterol level facilitates the prognostication/risk stratification of patients with CVD. Similarly, HbA1c levels help determine the risk for cardiovascular deaths and all-cause mortality in 5-6% of non-diabetic asymptomatic patients with CVD [55]. High levels of HbA1c in asymptomatic middle-aged/young adults linearly correlate with their risk of heart failure, coronary heart disease, stroke, and MI [56]. Recent findings reveal a marked elevation in the incidence/risk of subclinical CVD, carotid intima-media thickness, coronary artery calcification, and left ventricular hypertrophy with every 1% elevation in glycated hemoglobulin [57]. They also emphasize the prognostication of CAD severity and adverse outcomes guided by baseline HbA1C elevation [58].

## Conclusions

The findings of this review paper strengthen claims concerning the risk stratification/prognostication potential of cardiovascular testing in the setting of asymptomatic CVD. Cardiovascular tests discussed include both invasive testing with risk of contrast side effects and non-invasive testings with minimal side effects. These tests have very high sensitivities in screening asymptomatic CVD patients. Although beneficial, there is reluctance in both patients and physicians to carry out these tests in this patient population because of potential procedure-associated side effects and lack of knowledge on the prognostic benefits of these tests. Although these tests are all beneficial, it is advocated that clinicians utilize the non-invasive test more frequently in asymptomatic CVD patients to minimize the risks of side effects. Carotid dopplers, ABI measurements, laboratory tests for HgbA1c, and cholesterol are all non-invasive tests that can provide a wealth of prognostic information with little to no side effects. More invasive and risk-associated tests such as coronary angiography can be reserved for asymptomatic patients with a very high-risk profile and on a case-to-case basis. Future studies are required to investigate and elaborate on the scope of testing needed for every patient. These studies would also be helpful to evaluate the risk of false negatives/positives following the scalable use of cardiovascular testing. The benefit of routine use of cardiovascular testing across asymptomatic patients is necessary to enhance their diagnostic utility in the early detection of cardiovascular disorders.
